# Complete Genome Sequence of Mycobacterium fortuitum subsp. *fortuitum* JCM 6387, a Type Strain of Human-Pathogenic Mycobacteria Showing Inducible Macrolide Resistance

**DOI:** 10.1128/mra.00060-22

**Published:** 2022-03-31

**Authors:** Mitsunori Yoshida, Hanako Fukano, Masato Suzuki, Yoshihiko Hoshino

**Affiliations:** a Department of Mycobacteriology, Leprosy Research Center, National Institute of Infectious Diseases, Tokyo, Japan; b Antimicrobial Resistance Research Center, National Institute of Infectious Diseases, Tokyo, Japan; University of Rochester School of Medicine and Dentistry

## Abstract

Mycobacterium fortuitum subsp. *fortuitum* is a rapidly growing mycobacterial species for which pathogenic features are unclear. Here, we report the complete genome sequence of the Mycobacterium fortuitum subsp. *fortuitum* type strain. This sequence will provide essential information for future comparative genome studies of this mycobacterium.

## ANNOUNCEMENT

Mycobacterium fortuitum subsp. *fortuitum* is a rapidly growing nontuberculous mycobacterium (NTM) and a member of M. fortuitum group (1–3). It is noteworthy that M. fortuitum subsp. *fortuitum* possesses an erythromycin resistance methylase (*erm*) gene [designated *erm*(39)] that induces macrolide resistance. In contrast, NTM in the same group, such as M. peregrinum and M. senegalense, do not have this gene function (4, 5). Here, we report the complete genome sequence of M. fortuitum subsp. *fortuitum* type strain JCM 6387.

M. fortuitum subsp. *fortuitum* strain JCM 6387^T^ (=ATCC 6841, =CIP 104534, =DSM 46621) was inoculated on 2% Ogawa media (Kyokuto, Tokyo, Japan) and incubated at 30°C for 5 days. Genomic DNA was extracted by a standard phenol-chloroform method (6, 7). Long-read data were obtained with the MinION platform (Oxford Nanopore Technologies, Oxford, UK) as follows. Approximately 1 μg of the genomic DNA was used for library preparation with a ligation sequencing kit (Q20+) (SQK-Q20EA). The library was sequenced using an R10.3 flow cell (FLO-MIN111). Raw sequence data were base-called using Guppy version 5.0.16 with the base-calling model for Q20+ chemistry (https://github.com/nanoporetech/rerio). Using NanoFilt software (8), we trimmed the first 75 bp of each read and filtered the trimmed reads with quality scores of less than 12 or shorter than 1,000 bp. The remaining reads (458,025 reads and a read length *N*_50_ of 12,020 bp) were *de novo* assembled into one contig (6,485,838 bp) with the “suggestCircular” flag, using Canu version 2.2 (9) with following parameters: corOutCoverage, 1,000; ContigFilter, 50 10,000 1.0 0.5 50; and genomeSize, 6m. The nonredundant sequence of the contig (bp 44836 to 6450843) was extracted by SeqKit (10). Using the same DNA, Illumina paired-end (2 × 150-bp) reads were obtained with the MiniSeq system (Illumina, San Diego, CA). The DNA library was prepared using the Nextera XT DNA library kit. After checking the quality of raw reads (775,520 reads) using FastQC version 0.11.9 (http://www.bioinformatics.babraham.ac.uk/projects/fastqc/), these reads were mapped to the assembly using the BWA aligner version 0.7.17 (11) for sequence and assembly error correction with Pilon version 1.2.4 (12). Using DFAST version 1.4.0 with default setting (13–15), the polished assembly (6,406,072 bp, 66.2% G+C content, and 426-fold coverage) was subjected to taxonomic checks based on average nucleotide identity (ANI) values, gene annotation, and rotation to start with the *dnaA* gene.

The ANI value between JCM 6387^T^ and a reported draft genome sequence of JCM 6387 (GCA_000295855.1) was 99.93%. Also, the ANI values for M. fortuitum subsp. *acetamidolyticum* JCM6368 (GCA_001570465.1), *M. peregrinum* DSM 43271 (GCA_002102345.1), M. conceptionense CCUG 50187 (GCA_002102065.1), and M. boenickei JCM 15653 (GCA_010731295.1), which are the mycobacterial species phylogenetically closest to M. fortuitum subsp. *fortuitum* (16), were 98.75%, 88.13%, 87.11%, and 87.05%, respectively. The numbers of predicted coding sequences, rRNA operons, and tRNAs in the genome were 6,171, 6, and 65, respectively. We confirmed that JCM 6387^T^ harbors the *erm*(39) gene (located between bp 1817406 and 1818146 of the chromosome). The complete genome sequence of JCM 6387^T^ comprises important data for future comparative genome studies.

### Data availability.

The genome sequence and annotations of M. fortuitum were deposited at DDBJ/EMBL/GenBank under the accession number AP025518. Raw sequence data for strain JCM 6387^T^ were deposited under DRA accession number DRA013323.
